# Supplementary material for: The GA4GH Categorical Variation Representation Specification: A Unified Computational Framework for Reasoning over Genomic Variant Categories
Source: bioRxiv. 2026 Apr 30:2026.02.10.705161. Preprint. [Version 2] doi: 10.64898/2026.02.10.705161 (PMC13142434; doi:10.64898/2026.02.10.705161)
Supplement: Supplement 2 [file NIHPP2026.02.10.705161v2-supplement-2.pdf]

bioRxiv preprint doi: https://doi.org/10.64898/2026.02.10.705161; this version posted April 30, 2026. The copyright holder for this preprint (which was not certified by peer review) is the author/funder, who has granted bioRxiv a license to display the preprint in perpetuity. It is made available under aCC-BY 4.0 International license.

Supplementary Information

Supplementary Table 1

Supplementary Table 1: Landscape of resources relevant to categorical variation. A summary of genomic knowledgebases, clinical resources, and data models that store or use categorical variants, or that would benefit from interoperability with Cat-VRS.

Supplementary Fig. 1

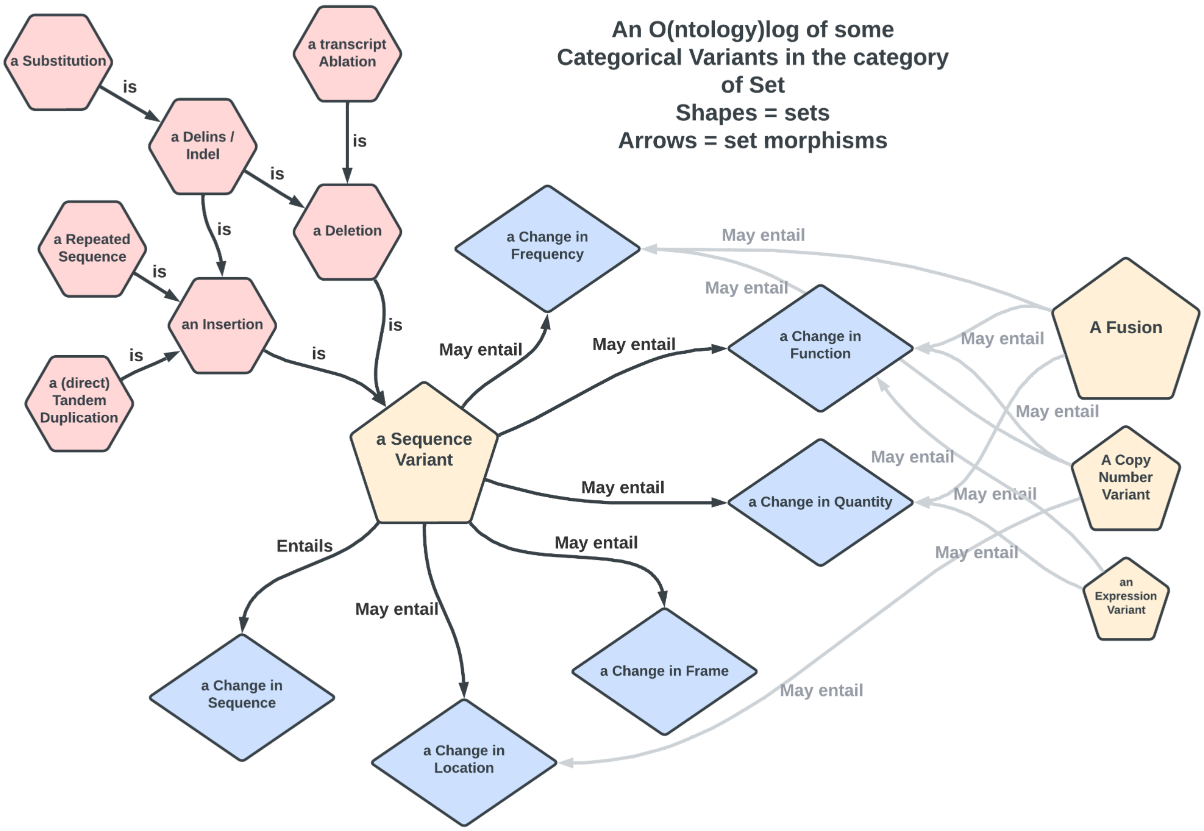

Supp. Fig. 1: Olog representation of CIViC categorical variant types.
This ontology log (olog) depicts a subset of CIViC's categorical variant types as set objects, with arrows representing definitional relationships (set morphisms) among them. By formalizing these categories and the properties they entail, the olog clarifies how CIViC's variant types relate to one another and salient semantic properties. This structural analysis provided the basis for identifying the minimal properties that govern category membership and informed the development of the typology and constraint-based representation used in Cat-VRS.
